# Active Targeting of Colorectal Cancer Using Chemotherapy-Loaded Nanoparticles Functionalized with a Folate Receptor-α (FRα) Ligand, Pemetrexed

**DOI:** 10.1007/s11095-025-03940-1

**Published:** 2025-11-18

**Authors:** Mohammad Alnatour, Ramkrishna Sen, Leela Sai Lokesh Janardhanam, Md Meraj Anjum, Sean M. Geary, Aliasger K. Salem

**Affiliations:** 1 Department of Pharmaceutical Sciences and Experimental Therapeutics, College of Pharmacy, University of Iowa, 452 CPB College of Pharmacy Building (CPB), 180S. Grand Avenue, Iowa City, IA 52242 USA; 2 Holden Comprehensive Cancer Center, University of Iowa, Iowa City, IA 52242 USA

**Keywords:** colorectal cancer, folate receptor-α (FRα), nanoparticles, pemetrexed, tumor-targeted drug delivery

## Abstract

**Purpose:**

Colorectal cancer (CRC) is a leading cause of cancer mortality with current chemotherapeutic strategies often limited by systemic side effects and suboptimal tumor targeting. This study aimed to enhance the delivery and efficacy of paclitaxel (PTX) for CRC therapy by engineering nanoparticles (NPs) actively targeted to the folate receptor-α (FRα) using pemetrexed, an FDA-approved antifolate with high FRα affinity.

**Methods:**

A novel FRα-targeted polymer (PLGA-PEG-pemetrexed) was synthesized by ring-opening polymerization and used to prepare tumor-targeted nanoparticles (TTNPs). The physical characteristics of TTNPs and non-targeted NPs (NTNPs) were evaluated by dynamic light scattering and TEM. Cellular uptake was assessed in FRα-expressing CT26 colorectal cancer cells by flow cytometry and confocal imaging. Cytotoxicity was evaluated using PrestoBlue™ assays. *In vivo* tumor targeting and therapeutic efficacy were assessed in a syngeneic CT26 tumor-bearing BALB/c mouse model using IVIS imaging, tumor accumulation and growth measurements.

**Results:**

The synthesized PLGA-PEG-pemetrexed formed uniform, negatively charged NPs with a hydrodynamic diameter of 140–170 nm. TTNPs demonstrated significantly enhanced uptake in FRα-expressing CT26 cells compared to NTNPs, which was abrogated by folic acid pre-treatment. *In vitro*, PTX-loaded TTNPs exhibited greater cytotoxicity against CT26 cells than free PTX. *In vivo*, TTNPs showed superior tumor accumulation compared to NTNPs, resulting in significantly greater tumor growth inhibition and increased intratumoral PTX concentrations. All treatments were well tolerated.

**Conclusion:**

Our results demonstrate that active targeting of chemotherapy-loaded NPs with a FRα ligand, pemetrexed, enhances tumor targeting and antitumor efficacy in a CRC model.

**Supplementary Information:**

The online version contains supplementary material available at 10.1007/s11095-025-03940-1.

## Introduction

Colorectal cancer (CRC) is the third most frequent cancer and the third most frequent cause of cancer deaths in the United States. Yearly, approximately 152,000 individuals are diagnosed with CRC and approximately 53,000 patients die from the disease [1]. This indicates that the current treatment approaches for CRC are still far from adequate and better treatment strategies need to be developed. Currently, CRC management involves surgery, radiotherapy, chemotherapy and targeted small molecule therapy. The first- and second-line chemotherapy treatment regimens often involve combinations of 5-fluorouracil (5-FU) and oxaliplatin (OX), or 5-FU and irinotecan. These pharmacological agents have demonstrated efficacy in treating a variety of solid malignancies, including CRC, however, they have serious side-effects including neurosensory toxicity, gastrointestinal toxicities, and neutropenia [2]. Systemic administration of chemotherapeutics packaged into nanoparticles (NPs) is considered a promising cancer-targeting approach that can reduce the side effects of chemotherapeutic drugs and enhance their effectiveness [3, 4]. Although NPs are renown for passively accumulating in solid tumors using the enhanced permeability and retention (EPR) effect, this property has proven ineffective at exacting extended survival in cancer patients. Recent studies have shown that even in high-EPR xenografted tumors, only a small fraction of NPs accumulate (less than 1%, according to a recent meta-analysis) at the tumor site [5, 6]. This could be due to several factors including the abnormal tumor vasculature, high interstitial pressure, tumor growth-induced solid stress, and solid stress from the abnormal matrix [7]. In addition, NPs are often taken up by other organs possessing sinusoids such as the spleen and liver where they become physically trapped and cleared by the mononuclear phagocytic system [8]. Hence, active tumor targeting is required to ensure improved drug accumulation and consequently enhanced antitumor activity at the tumor site. This can be achieved by grafting tumor targeting moieties onto the NP surface [9, 10]. In CRC, the overexpression of specific cell surface molecules is a common occurrence. These include the cell adhesion protein CEA, the tumor-associated glycoprotein-72 (TAG-72), the folate receptor-α (FRα), and the epithelial growth factor receptor (EGFR). Comparative analysis with matched healthy tissues revealed their overexpression in 98.8%, 79.0%, 37.1%, and 32.8% of cases, respectively [11]. CEA, TAG-72 and EGFR can only be targeted using polypeptides, aptamers and antibodies, and such labile targeting moieties are usually added to the NP surface post formulation, [12–14]. On the other hand, FRα can be targeted using small molecules (folic acid and folate analogues) that can be linked to biocompatible polymers and used to prepare tumor targeted formulations in one step. In addition, FRα upregulation is associated with poor prognosis, and worse survival in patients with stage 4 CRC [15, 16]. These findings make FRα a promising target for metastatic and drug-resistant CRC. Folic acid has been repeatedly used to target folate receptors with some success, but as yet none of the developed formulations have progressed to clinical trials. Pemetrexed (Alimta™) is an FDA-approved antifolate cytotoxic agent with a two-fold higher affinity for FRα compared to folic acid [17, 18]. Utilizing pemetrexed as a targeting moiety for FRα could potentially yield superior outcomes compared to nontargeted NPs. Thus here, for the first time we show that covalently attaching pemetrexed to PLGA during de novo polymer synthesis and then incorporating the conjugated drug-polymer into NPs enhances tumor-targeting properties.

Paclitaxel (PTX) is a chemotherapeutic agent marketed under the brand name Taxol. Used as a treatment for various cancers, PTX is a microtubule depolymerization inhibitor that has been in clinical use for decades. Currently, PTX is not FDA-approved for treating CRC and this appears to be based on a single clinical trial performed almost 30 years ago on a limited number of CRC patients (*n* = 16) who were treated with Taxol [19]. The study was compromised by the high-grade neutropenia limiting the delivered dose. Since then, with the advances in nanotechnology and the understanding of mechanisms of drug resistance and how to overcome them, PTX has regained interest as a potential treatment, or adjuvant to current treatment strategies, for CRC [20].

In the present work, PEG 2000-pemetrexed was used as an initiator for ring-opening polymerization (ROP) reaction of glycolide and lactide, resulting in the synthesis of PLGA-PEG-pemetrexed polymers (PLGA-P). The resultant polymers demonstrated the ability to self-assemble into NPs encapsulating fluorescent probes and chemotherapeutic drugs.* In vitro* investigations revealed a significantly higher uptake of FRα-targeted NPs by CRC cell lines expressing the FRα. The receptor-dependent nature of this uptake was further validated through receptor-blocking experiments. PTX was chosen to develop cytotoxic tumor targeted NPs (TTNPs) over other commonly used chemotherapeutics in CRC research for its ideal physicochemical properties that make it easy to formulate into polymeric NPs with superior drug loading over more conventional first line therapy agents, OX and 5-FU, used to treat CRC.* In vivo* studies involving BALB/c mice challenged with the syngeneic CRC cell line, CT26, demonstrated that the PTX-loaded TTNPs exhibited significantly greater anti-tumor activity and increased delivery of PTX to tumors compared to PTX-loaded NTNPs.

## Materials and Methods

### Reagents

Lactide, glycolide 99%, coumarin-6, tin(II) 2-ethylhexanoate (Sn(Oct)_2_) 92.5–100.0%, D-α-tocopherol polyethylene glycol 1000 succinate (TPGS), folic acid (FA), 5-fluorouracil (5-FU) and deuterated dimethyl sulfoxide (DMSO-d6) were purchased from Sigma-Aldrich (St. Louis, MO). DiR, oxaliplatin and docetaxel were purchased from MedChemExpress (Monmouth Junction, NJ). Poly (D, L-lactide-co-glycolide) (PLGA: MW = 20 kDa)) methoxy polyethylene glycol (mPEG: MW = 2 kDa) was purchased from BroadPharm (San Diego, CA). Pemetrexed-PEG-OH was purchased from Biochempeg Scientific (Watertown, MA). Paclitaxel powder was purchased from LC Laboratories (Woburn, MA), Taxol (6 mg mL^−1^, Cat. No. 70860–200-05) for intravenous (IV) administration was purchased from Athenex, Inc. (Buffalo, NY). Dichloromethane (DCM), tert-Butyl methyl ether (tBME) acetonitrile (ACN) and methanol were HPLC grade provided by Fisher Scientific (Waltham, MA). CT26 cells were purchased from ATCC (Manassas, VA) and were maintained in RPMI 1640 Medium, supplemented with 2 mM L-glutamine, 1 mM sodium pyruvate, 10 mM HEPES and 100 µM gentamicin, all obtained from Gibco (Grand Island, NY), and 10% fetal bovine serum was purchased from Bio-Techne (Flowery Branch, GA). All the materials and solvents were used as received unless stated otherwise.

### Polymer Synthesis

**Fig. 1:**
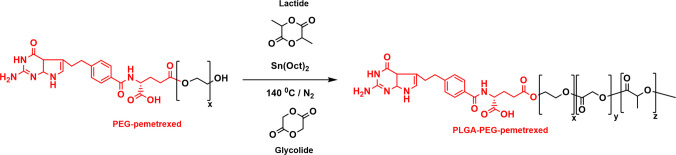
Schematic representation of ROP reaction of lactide and glycolide in the presence of PEG-pemetrexed as initiator.

The PLGA-PEG-pemetrexed polymer was synthesized by ring-opening polymerization (ROP) of lactide and glycolide using PEG-pemetrexed as the initiator as shown in Fig. 1. PEG-pemetrexed (0.5 g, 0.2 mmol), lactide (1.441 g, 10 mmol) and glycolide (1.161 g, 10 mmol) were melted at 140°C under a nitrogen atmosphere. Sn(Oct)_2 _(0.5% wt/wt) was added to the reactant mixture and the reaction continued for 12 h. Thereafter, the reaction mixture was cooled to room temperature. The synthesized PLGA-PEG-pemetrexed was purified by dissolving in DCM and precipitating in ice-cold methanol three times to remove remaining unreacted monomers, PEG-pemetrexed, reaction catalyst, and short polymer chains.

^1^H-NMR spectroscopy was performed using a Bruker Avance NEO 500 NMR instrument to confirm the synthesized polymer chemical structure and molecular weight. Briefly 10 mg of the polymer was dissolved in DMSO-d_6_ as a solvent and then the sample was transferred to a NMR tube using a Pasteur pipette. All the chemical shifts were recorded in ppm. The spectra were analyzed using Bruker Topspin software (TopSpin 4.3.0).

### Nanoparticle Preparation and Characterization

Using PLGA-PEG-pemetrexed, TTNPs were prepared by a previously described single emulsion method with some amendments [21]. Briefly, 20 mg of the PLGA-PEG-pemetrexed was dissolved in 1 ml of DCM to form the oil phase, and 10 ml of 1% (w/v) TPGS solution was used as the aqueous phase. Upon combining, the two phases were sonicated at 90% amplitude for 5 min using a Qsonica probe sonicator (Q500 Sonicator) in an ice-cold water bath. After that, the mixture was stirred at 300 rpm for three hours using a magnetic stirrer in a fume hood to allow the DCM to evaporate. Then the NPs were purified by dialyzing against two liters of deionized water for 6 h using a 1000 kDa Cellulose Dialysis Membrane, (Spectra/Por; Waltham, MA). Non-targeting NPs (NTNPs) were prepared following the same procedure instead substituting PLGA-PEG-pemetrexed with commercially available PLGA-PEG. PTX-loaded and fluorescently-labeled NPs were prepared using the aforementioned procedure by adding 6 mg PTX, 20 µg coumarin-6 (C-6) or 400 µg DiR to the DCM polymer solution.

### Particle Size and Zeta Potential Measurements

Mean hydrodynamic diameters and zeta potential values of the prepared NPs were obtained by measuring the light scattering at 173° angle to incident radiation at 25°C using a Malvern (TM) Zetasizer (Westborough, MA; equipped with 10 mW He–Ne laser operating at a wavelength of 633 nm) after diluting the samples with deionized water.

### High-performance Liquid Chromatography

In order to determine PTX encapsulation, high-performance liquid chromatography (HPLC) (Waters Milford, MA, USA) was used for the determination of nanoparticle-encapsulated PTX. First, 1 mg of the experimental sample was dissolved in 1000 μL of ACN, and 10 μL of the resulting supernatant was injected into a Kinetex C18 column (4.6 mm × 150 mm, particle size 5 μm, Phenomenex, Torrance, CA, USA) maintained at 30°C. A mobile phase consisting of 0.1% formic acid in water (A) and acetonitrile (B) was run isocratic for 6 min at a flow rate of 1 mL/min as follows: 45% A, 55% B, and the detection wavelength was 548 nm. The drug content in the drug-loaded nanoparticles was calculated from the standard curve. The encapsulation rate was calculated by the following equation. Encapsulation Efficiency (%) = amount of paclitaxel in the nanoparticles/total drug added × 100%. Drug loading (%) = amount of paclitaxel in the nanoparticles/total nanoparticles weight × 100%.

### Transmission Electron Microscopy (TEM)

NP morphology was evaluated by using Hitachi HT-7800 Transmission Electron Microscopy (TEM). Seventeen µl of NPs suspended in water at 2 mg/ml were pipetted onto a copper grid and wicked off after 10 min; subsequently, the grid was negatively stained with 3% w/v of uranyl acetate for 2 min prior to image acquisition and analysis.

### Cellular Uptake Studies

Cells were seeded into 24 well plates or Nunc™ Lab-Tek™ 4-Chamber Slide System (Thermo Fisher Scientific) (coverslip bottom) at a concentration of 50,000 cells per well/chamber (37°C, 5% CO_2,_ and 95% humidity) for 48 h. Then the media was substituted with 0.5ml media containing 100 μg/mL of fluorescently (coumarin-6)-loaded TTNPs or NTNPs (37°C, 5% CO_2,_ 1 h). Following treatment, cells in the 24 well plates were washed twice with prewarmed PBS, trypsinized and analyzed by flow cytometry (FACScan, BD Biosciences) after quenching and washing away trypsin. For confocal imaging (where chamber slides were used), cells were washed twice with PBS and then stained with CellMask™ Plasma Membrane Stains, deep red 5 µg/ml for 5 min then washed twice with PBS and then fixed with 4% paraformaldehyde for 15 min at room temperature, washed three times for 5 min/wash with PBS. Finally, cells were stored with mounting media (VECTASHIELD® PLUS Antifade Mounting Medium with DAPI, 4°C) ready for imaging. For FRα blocking studies, following a previously published protocol [22], saturating levels (1 mM) of FA were added to the cell cultures two hours prior to the addition of NPs.

### *In Vitro* Cytotoxicity Toward CT26 Cells

The PrestoBlue™ metabolic activity assay was used to assess cell viability. CT26 cells were seeded on 96-well plates at a concentration of 5,000 cells/well for 24 h. PTX (dissolved in DMSO), blank TTNPs, PTX-loaded TTNPs and PTX-loaded NTNPs were added to cells and the plates were incubated for 48 h. Thereafter, 10 µl of PrestoBlue™ Cell Viability Reagent (Thermo Fisher Scientific) was added to each well and incubated for 10 min (37°C, 5% CO_2_ and 95% humidity). The fluorescence was recorded (SpectraMax M5, Molecular Devices, Sunnyvale, CA, USA) at excitation/emission of 560/590 nm. The results were plotted as mean % viability vs control ± standard deviation (SD). Positive controls (cells treated with triton- × 1% v/v) and negative controls (untreated cells) were used to assess the results including additional controls: cells treated with blank NPs, and cells treated with PTX in soluble form. Percentage cell viability was expressed as the average fluorescence of the test group relative to that of the control group (untreated cells).

The PrestoBlue™ metabolic activity assay was also used to determine the most effective anti-cancer agent. Briefly, CT26 cells were seeded on 96-well plates at a concentration of 5,000 cells/well for 24 h and then the media was replaced with fresh media containing 10,000, 1000, 100, 10, 1, 0.1, 0.01 nM of 5-FU, PTX or OX and incubated for 48 h (37°C, 5% CO_2_ and 95% humidity). Thereafter Prestoblue™ reagent was added and viability was determined as described above.

### *In Vivo* and *Ex Vivo* Imaging of NP Accumulation in CT26 Bearing BALB/c Mice

All animal procedures were approved by the Institutional Animal Care and Use Committee (IACUC). Six-eight week-old female BALB/c mice were inoculated subcutaneously with 3 × 10^5^ CT26 cells/mouse on the rear right flank and the tumor volumes were recorded every 2 days. Once the tumors reached ∼400 mm^3^, the mice were randomly assigned to three different treatment groups (*n* = 3) and received a single tail vein injection dose (4 mg NPs per mouse) of DiR-loaded TTNPs, DiR-loaded NTNPs, or blank TT NPs. Whole body images were acquired under 2% isoflurane anesthesia at specified times post-injection of indicated formulation using a PerkinElmer IVIS® Lumina™ S5 Imaging System (Waltham, MA). After the final imaging (96 h), animals were humanely sacrificed, and the tumors were collected and imaged by IVIS. Total Radiant Efficiency (µW/cm^2^) of a region-of-interest (ROI) was acquired by the Living Image™ Software following a previously published protocol [23].

### *In Vivo *Efficacy Studies Using a Syngeneic Model of CT26 Bearing BALB/c Mice

All animal procedures were approved by IACUC. Six-eight week-old female BALB/c mice were inoculated subcutaneously with 3 × 10^5^ CT26 cells/mouse on the rear right flank and the tumor volumes were recorded every 2 days. After the tumors reached ∼100 mm^3^ in size, the mice were randomly assigned to four different treatment groups (*n* = 8/group): Group 1: PTX-loaded TTNPs; Group 2: PTX-loaded NTNPs; Group 3: Taxol; Group 4: 100 µl of normal saline. All treatments were delivered via a tail I.V. injection (100 µl) equivalent to 10 mg/kg of PTX on day 0 and 7. The tumor diameters and height were measured using digital calipers. The tumor volumes were calculated from the following formula:

$$Tumor\;volume\;\left(mm^3\right)=\frac\pi6\times D_1\times D_2\times H$$

where D_1_ and D_2_ are the first and second tumor diameters (mm), respectively and H is the height (mm). Mice weights were monitored during the experiment and mice were euthanized once the tumor diameter exceeded 20 mm in any direction.

### Biodistribution of Systemically Administered TTNPs in CT26 Bearing BALB/c Mice

All animal procedures were approved by IACUC. Six-eight week-old female BALB/c mice were inoculated subcutaneously with 3 × 10^5^ CT26 cells/mouse on the rear right flank and the tumor volumes were recorded every 2 days. After the tumors reached ∼500–750 mm^3^ in size, the mice were randomly assigned to two treatment groups (*n* = 12/group): Group 1: PTX-loaded TTNPs and Group 2: Taxol. Each group received one dose equivalent to PTX 10 mg/kg. After that 3 mice from each group were sacrificed and major organs (liver, spleen, heart, kidney, lung) and tumors were collected to estimate PTX concentration at the following time points: 1, 4, 24 and 48 h. The collected organs were homogenized in ice-cold PBS (0.3 g tissue/mL PBS) using a Tissue-Tearor homogenizer (Bartlesville, Oklahoma). Each homogenate (0.35 mL) was spiked with 600 ng of docetaxel (DTX) as internal standard and mixed with 1.5 mL of methyl *tert*-butyl ether (tBME). The mixture was vortexed for 5 s for PTX extraction and rotated for 1.5 h. After centrifugation at 10 000 rcf for 10 min, 1 mL of the tBME phase was collected, evaporated, and redissolved in a 60 μL of ACN for LC/MS analysis. Calibration standards were prepared by adding PTX to respective tissue homogenates (0.35 mL) in known quantities (1000, 500, 250, 125, 62.5, 31.3 and 15.6 ng) and processed in the same way as samples. PTX quantification was performed with a Xevo TS-Q cronos LC/MS/MS mass spectrometer (Waters Corporation, Milford, MA, USA). Five microliters of samples or standards were injected onto a Kinetex C18 column (4.6 mm × 150 mm, particle size 5 μm, Phenomenex, Torrance, CA, USA) maintained at 30°C. A mobile phase consisting of 0.1% formic acid in water (A) and acetonitrile (B) was run isocratic for 10 min at a flow rate of 0.8 mL/min as follows: 45% A, 55% B. The parent ions, [PTX + H]^+^ and [DTX + H]^+^, were monitored at the *m*/*z* ratios of 854.2 and 808.3, respectively. Peak ratios of PTX to DTX were plotted against the nominal concentration of PTX to generate a calibration curve from each tissue.

### Statistical Analysis

All the *in vitro* experiments were repeated at least three times. Data are expressed as mean ± SEM. Statistical analysis was performed using GraphPad prism software for Windows version 10.0.0 (GraphPad Software, Inc., San Diego, CA). One-way analysis of variance (ANOVA) followed by Tukey post hoc test was used to compare between 3 or more groups and unpaired- *t*-test was used to compare between two groups. Differences were considered significant at *P* < 0.05.

## Results

### Polymer Synthesis

**Fig. 2:**
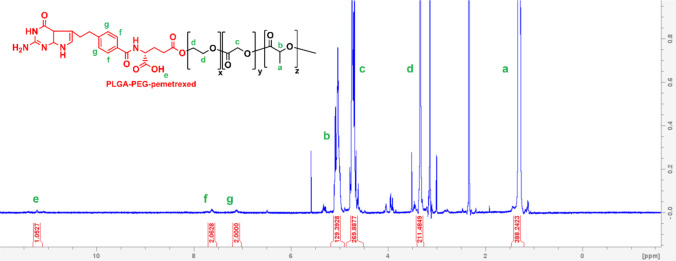
^1^H NMR spectrum of PLGA-PEG-pemetrexed in DMSO-d_6_, prepared by ROP with Sn(Oct)_2_ as catalyst and PEG-pemetrexed as initiator. Green letters annotate each peak to its corresponding protons and the red numbers at the bottom of the figure represent the integration (area under the peak)

A pemetrexed-terminated PLGA-PEG copolymer was successfully synthesized by ROP of lactide and glycolide using PEG-pemetrexed as an initiator in the presence of the catalyst Sn(Oct)_2_. Briefly, the terminal hydroxyl group at the end of OH-PEG-pemetrexed reacted with a carbonyl group in the lactide or a glycolide molecule resulting in freeing another hydroxyl group and the reaction continued until all the glycolide and lactide were depleted. The resultant polymer was given the name PLGA-P (= PLGA-PEG-pemetrexed), and the polymer formation was validated by NMR spectroscopy (Fig. 2). Table 1 shows the characteristics of PLGA-P with a yielding percent of 71%.

**Table I:** Characteristics of the synthesized PLGA-P polymer

Polymer	Theoretical monomer ratio (mol %)			Actual monomer ratio (mol %)			Theoretical and actual polymer molecular weight (g mol^−1^)			M_w/_Mn	Yield (%)
	LA	GA	PEG-P	LA	GA	PEG-P	M (calc)	M_n_	M_w_		
PLGA-P	50	50	1	48.5	50.75	0.75	19,716	11,778	14,365	1.22	71%

### NP Preparation and Characterization

**Fig. 3:**
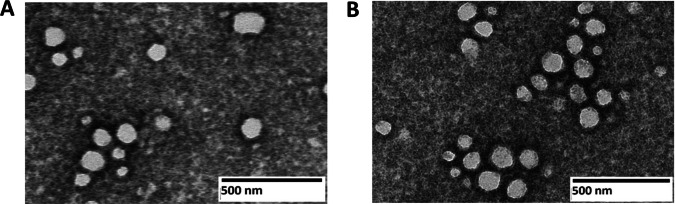
TEM images of TTNPs (**A**) and NTNPs (**B**) where scale bar = 500 nm

PLGA-P and the commercially available PLGA were used to synthesize TTNPs and NTNPs, respectively, using a single emulsion method. Both TTNPs and NTNPs displayed comparable hydrodynamic diameters of 140–170 nm, low PDIs (< 0.25) and zeta potentials of approximately −19 to −23 mV (Table 2). TEM images (Fig. 3A and B), indicated low size dispersity and there were no discernible morphological differences between TTNPs and NTNPs. These findings align with previously published literature for PLGA-based NPs [21, 24–26].

**Table II:** Formulation parameters, composition, size, zeta potential, and encapsulation efficiency, drug loading of the NPs made from PLGA-PEG-pemetrexed or PLGA-PEG polymers: blank, coumarin-6-(C6)-loaded or PTX-loaded NPs were analyzed. *PDI* = polydispersity index

Formula code	Drug	PLGA-P (mg)	PLGA- PEG (mg)	Organic phase (ml)	Aqueous phase (ml) 1% TPGS	Sonication time (min)	Hydrodynamic diameter (nm)	PDI	Zeta potential (mV)	Encapsulation efficiency	Drug Loading
NTNPs (blank)	–-	–-	20	1 DCM	10	5	138.4 ± 9.3	0.22	−22.2 ± 1.4	–-	–-
TTNPs (blank)	–-	20	–-	1 DCM	10	5	144.5 ± 7.4	0.13	−19.2 ± 2.1	–-	–-
C6 NTNPs	20 µg C6	–-	20	1 DCM	10	5	156.5 ± 10.6	0.12	−21.3 ± 1.6	–-	–-
C6 TTNPs	20 µg C6	20	–-	1 DCM	10	5	168.3 ± 12.4	0.2	−22.3 ± 2	–-	–-
PTX NTNPs	6 mg PTX	–-	20	1 DCM	10	5	162.3 ± 17.4	0.22	−23.7 ± 3.1	55.4 ± 4.7%	18.1 ± 1.7%
PTX TTNPs	6 mg PTX	20	–-	1 DCM	10	5	169.4 ± 15.7	0.15	−21.6 ± 2.2	57.2 ± 5.4%	17.7 ± 2%

### Cellular Uptake Studies

**Fig. 4:**
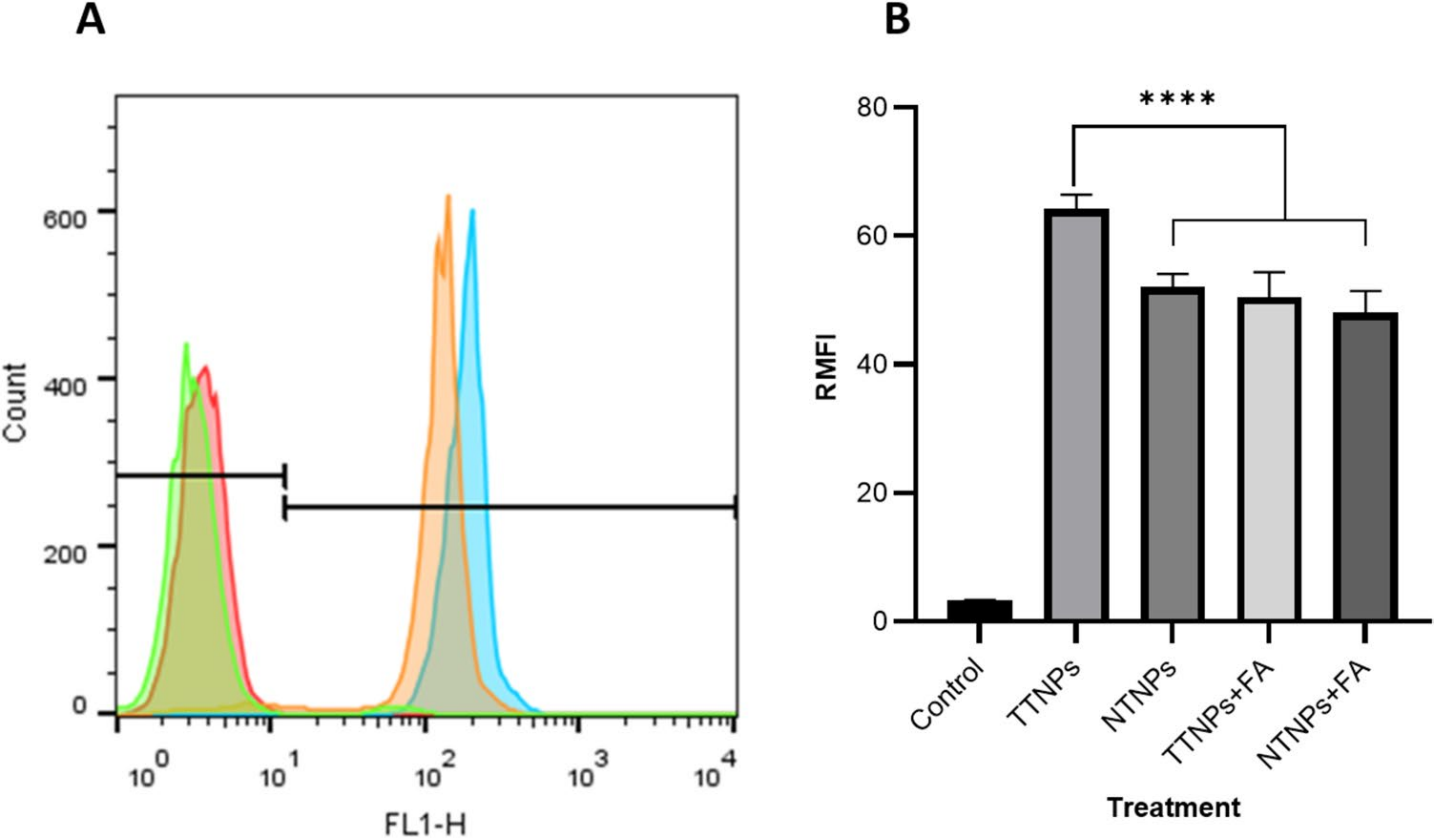
CT26 uptake of TTNPs versus NTNPs: **A**) Flow cytometer derived histogram overlays of CT26 cells treated with coumarin-6 (C6)-loaded TTNPs (C6-TTNPs) (blue), C6-NTNPs (orange), and blank-TTNPs (pink/red) at a concentration 100 µg/mL for 1 h: untreated control (green). **B**) Flow cytometric relative median fluorescence intensities (RMFI) of CT26 cells exposed to C6-TTNPs and C6-NTNPs. The exposure was carried out with and without two-hour pretreatment with 1 mM FA. *n* = 4/group, one-way ANOVA, * *p*-value < 0.05 and ** *p*-value < 0.01, **** *p*-value < 0.0001

CT26 is a mouse CRC cell line known to express the FRα and thus was used to test the active targeting properties of our TTNPs *in vitro* [27]. Semiquantitative determination of uptake of fluorescently labeled NPs was carried out using cell culture and flow cytometry as described in the *methods* section. CT26 cells were cultured with either C-6-loaded TTNPs or C-6-loaded NTNPs for 1 h and then the cells were harvested and run through the flow cytometer and analyzed to compare uptake rates. TTNPs were significantly more taken up by CT26 cells compared to NTNPs (Fig. 4A and B). To ensure the enhanced uptake was FRα-specific, CT26 cells were pretreated with 1 mM FA for two hours to block FRα, and it was observed that there was no significant difference in uptake between the TTNPs and NTNPs in the presence of FA (Fig. 4B). Similar results were obtained from FRα receptor-expressing human colorectal cancer cells, SW620 and no preferential uptake was noticed in FRα non-expressing human CRC cell line HCT116, Figure S1.

**Fig. 5:**
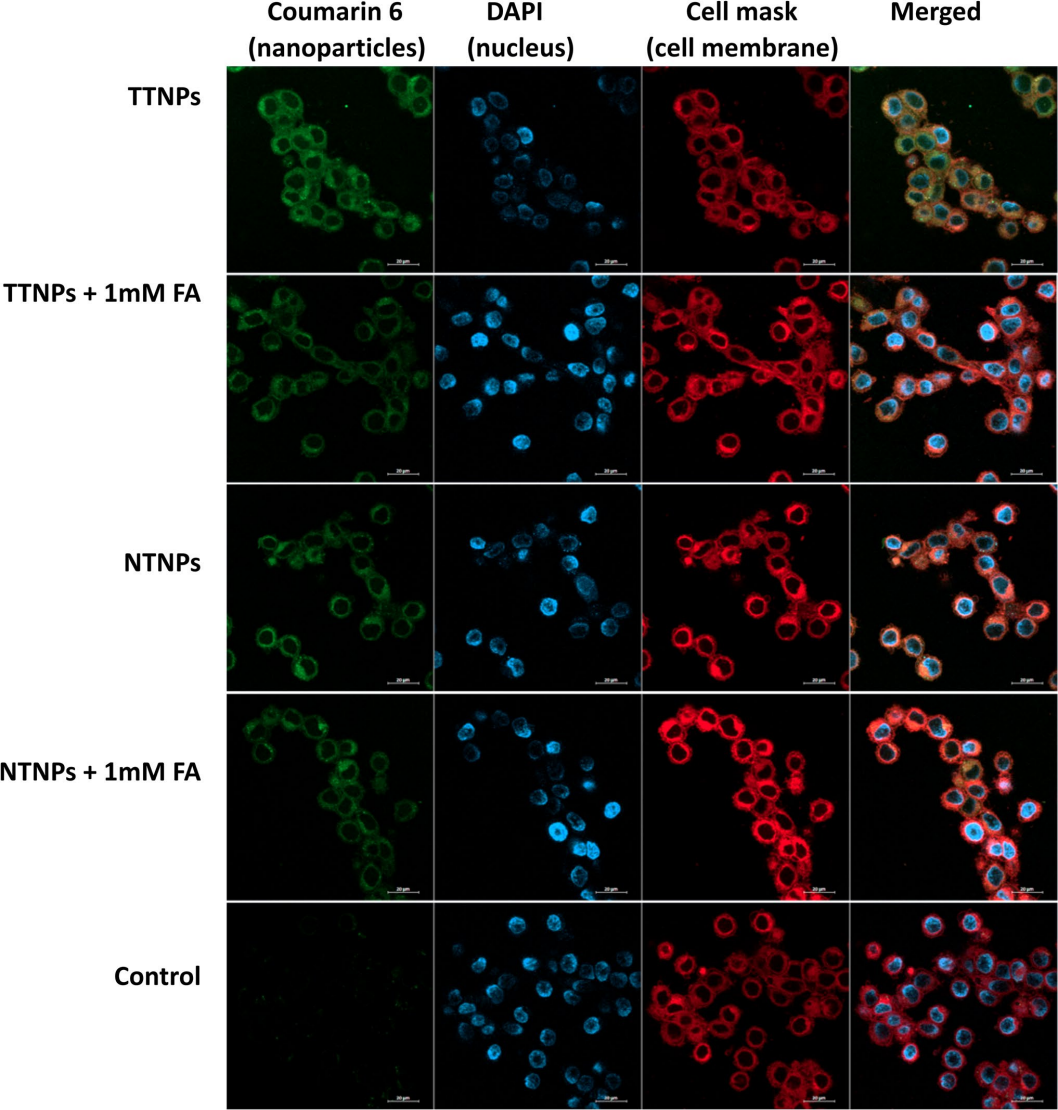
Confocal imaging of CT26 cells exposed to 100 μg/ml of C6-TTNPs and C6-NTNPs with or without pretreatment with 1 mM FA for 1 h. NP uptake (green); DAPI-stained nuclei (blue); cell membrane stained with CellMask™ Deep Red (red)

The intracellular location of internalized particles was investigated using wide-field confocal microscopy. CT26 cells were seeded into coverslip bottom chamber slides and subsequently dosed with TTNPs and NTNPs at (100 µg/mL) for 1 h and imaged using confocal microscopy to confirm their intracellular localization as opposed to merely attaching to the cell surface (Fig. 5).

### *In vitro *Antitumor Assessment

**Fig. 6:**
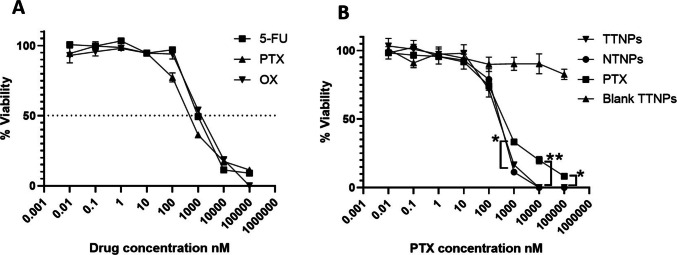
Cell viability assays. **A**) Dose–response curves of CT26 cells treated with indicated concentrations of 5-FU, PTX or OX for 48 h; data are presented as mean ± SEM. **B**) Dose–response curves of CT26 cells treated with PTX-loaded TTNPs, PTX-loaded NTNPs, soluble PTX at indicated concentrations of PTX or Blank TTNPs at equivalent dose of PTX loaded TTNPs; data are presented as mean ± SEM. *n* = 3/group, ** *p*-value < 0.01, **** *p*-value < 0.0001 using one-way ANOVA

The ability of TTNPs to deliver chemotherapeutic agents to FRα expressing cells was assessed using a cell metabolic activity assay. CT26 cells were exposed to an increasing concentration of three commonly used chemotherapeutic drugs in CRC research; 5-FU, PTX and OX. PTX demonstrated the lowest IC_50_ and was subsequently used for the NP formulations (Fig. 6A). CT26 cells were exposed to PTX-loaded TTNPs, PTX-loaded TTNPs and free PTX. TTNPs and NTNPs were able to enhance the cytotoxicity of PTX (versus soluble PTX) at concentrations higher than 1000 nM (Fig. 6B).

### *In vivo* and *Ex Vivo* Imaging of NP Accumulation in Tumor-Bearing Mice

**Fig. 7:**
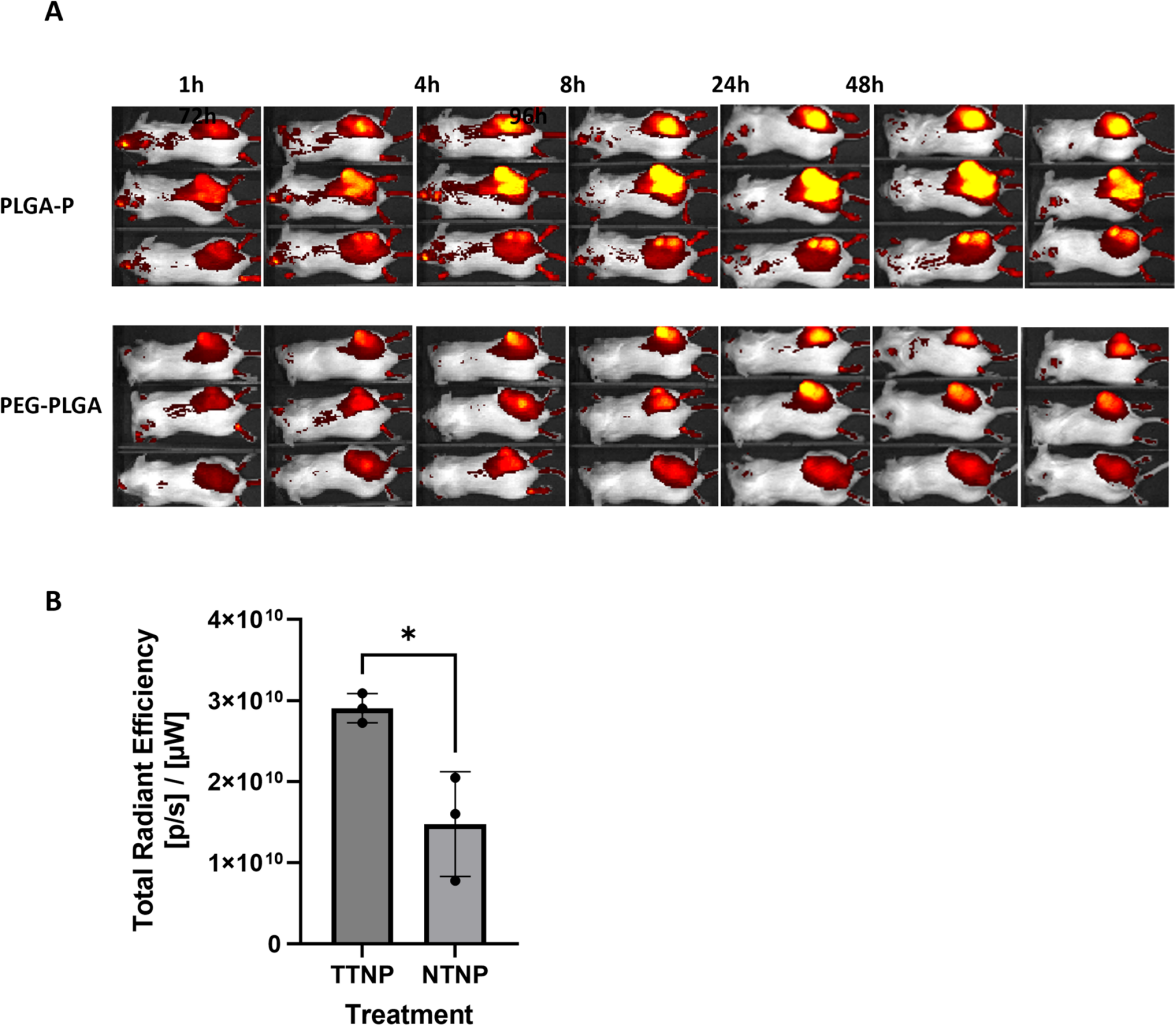
**A** Real-time whole-body imaging of CT26 tumor-bearing female BALB/C mice at different time points after tail vein injection of DiR PLGA-P NPs and DiR PEG-PLGA NPs at a dose of 4 mg NPs per mouse; dorsal view. **B** Ex vivo quantitative image analysis of tumor by total radiant efficiency normalized to tumor surface area, *n* = 3/group; t-test was used to obtain statistical significance; * *p-*value < 0.05

The *in vivo* tumor accumulation of DiR-loaded TTNPs and DiR-loaded NTNPs were compared in BALB/c mice bearing subcutaneous CT26 tumors (Fig. 7A). In general, TTNPs showed greater fluorescence intensity in CT26 tumors than NTNPs, this was expected from the *in vitro* study. Ex vivo tumor DiR signal was used to assess relative NP accumulation; comparing DiR-loaded TTNPs to DiR-loaded NTNPs, revealing TTNPs to have accumulated in the tumor to a significantly greater extent than NTNPs (Fig. 7B).

### *In Vivo *Efficacy Studies Using a Syngeneic Model of CT26 -Challenged BALB/c Mice

**Fig. 8:**
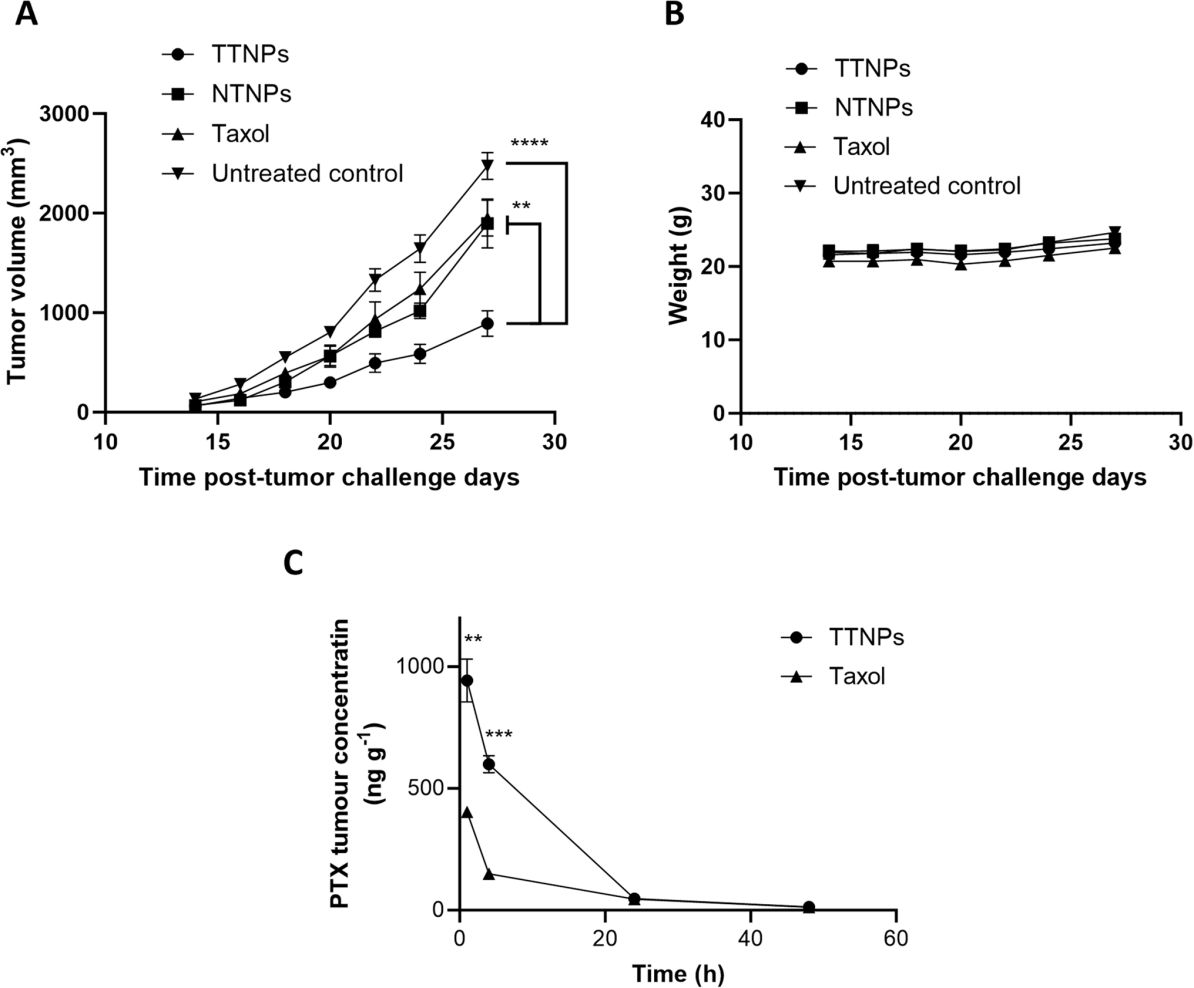
**A** Tumor growth profiles for CT26 tumor-bearing BALB/C mice, I.V. injected with PTX-loaded TTNPs, NTNPs, Taxol, or blank TTNPs. *N* = 8/group. Two doses of PTX/Taxol equivalent to 10 mg/kg on Day 0 and Day 7. Tumors were measured every other day. Statistical analysis was performed using a one-way ANOVA. **B** Mice weight change over time during treatments. Mice were weighed every other day. Data are presented as mean ± SEM. C) Intratumoural PTX concentration over a 48 h period after a single i.v. (tail vein) injection of either 10 mg kg–1 Taxol or 10 mg kg–1 TTNPs (eq PTX) quantified using a LC–MS/MS. Statistical analysis was performed using an unpaired two-tailed t-test. Data are expressed as mean ± SEM. (*n* = 3). **P < 0.01

BALB/c mice were challenged with CT26 cells and upon tumors reaching 100 mm^3^ mice were treated (I.V.) with PTX-loaded TTNPs, PTX-loaded NTNPs or taxol at 10 mg/kg PTX per mouse on days 0 and 7. Tumor volumes and body weights were recorded until day 27, when the experiment was terminated. Treatment with TTNPs was superior to NTNPs and Taxol in terms of slowing the rate of tumor progression, which indicates that the delivery of PTX in TTNPs improves efficacy (Fig. 8A). There was no significant change in mouse weights which suggests that all treatments including TTNP treatment were well tolerated by the mice (Fig. 8B). The enhanced efficacy is likely to be due to enhanced delivery of PTX to the tumor in terms of being present at the tumor site for longer and being there at higher concentrations as was seen in Fig. 8C as determined by LC–MS/MS.

## Discussion

In this study, we synthesized and evaluated PLGA-PEG-pemetrexed polymer-based nanoparticles (TTNPs) for the targeted delivery of PTX to CRC expressing FRα. PLGA-PEG-pemetrexed (PLGA-P) polymer was successfully synthesized via ROP, and confirmed by 1H NMR spectroscopy. The resulting polymer efficiently self-assembled into uniform NPs displaying favorable physicochemical properties essential for passive and active targeting [28]. Active targeting of NPs via FRα is particularly advantageous given its frequent overexpression in aggressive CRC subtypes and poor prognosis associated with elevated FRα expression[15, 16]. Pemetrexed, a clinically approved antifolate drug was strategically used because of its superior affinity for FRα compared to folic acid [17, 18]. This approach simplifies manufacturing processes by integrating the targeting ligand into the polymer backbone, avoiding the complications associated with post-formulation ligand attachment, such as changes in hydrodynamic diameter, changes in physical stability and limited functional group availability [29, 30].

Cellular uptake studies confirmed that TTNPs showed significantly higher internalization in FRα-expressing CT26 cells compared to non-targeted NPs (NTNPs). This enhanced internalization was specifically mediated through the FRα, as pre-blocking with excess folic acid diminished TTNP uptake, aligning with previously reported receptor-mediated uptake mechanisms [22, 27].

Although TTNPs demonstrated enhanced internalization relative to NTNPs (uptake studies (Fig. 4) and internalization (Fig. 5) *in vitro* as well as enhanced tumor accumulation (Fig. 7), drug delivery (Fig. 8C) and anti-tumor activity (Fig. 8A) *in vivo*), the cytotoxic efficacy of both NP formulations was comparable *in vitro*. This phenomenon can be attributed to the propensity of cancer cells to efficiently internalize NPs through non-specific endocytic pathways, irrespective of surface ligand decoration, especially under *in vitro* conditions where physiological barriers are absent and the cells being constantly exposed to NPs for long durations [31–33]. Furthermore, *in vitro* models may not adequately reflect the differences in biodistribution, clearance, and microenvironmental factors present *in vivo*, where active targeting has a greater impact [34]. Thus, while the targeting ligand (pemetrexed) confers specificity, its full therapeutic advantage is most evident *in vivo*, where TTNPs exhibited significantly superior tumor accumulation and efficacy compared to both NTNPs and free drug. The enhanced targeted accumulation of TTNPs in tumors is likely attributable to FRα-mediated endocytosis and retention within tumors, supporting previously established paradigms of folate active NP targeting [23].

*In vivo* therapeutic efficacy assessments demonstrated clear superiority of PTX-loaded TTNPs compared to PTX-loaded NTNPs and clinically used Taxol. Tumor growth was significantly impeded, correlating closely with increased intratumoral PTX concentrations as verified by LC–MS/MS analysis. Importantly, treatment regimens were well-tolerated with no adverse effects on body weight, underscoring the clinical translatability of the formulation. The observed therapeutic advantage highlights the importance of active targeting mechanisms in NP-based drug delivery. Previous reports suggest passive accumulation alone via the EPR effect is often insufficient to yield clinical success, reinforcing the necessity of actively targeted NPs [35]. Our approach demonstrates considerable promise in addressing unmet clinical needs in CRC management by combining active targeting and effective drug encapsulation strategies.

Future work should explore further optimization of the formulation parameters, long-term pharmacokinetic studies, and comprehensive toxicological assessments to translate this promising targeted NP formulation toward clinical trials. Additionally, combination therapies integrating immunotherapeutics or other anticancer agents with these targeted NPs might further enhance therapeutic efficacy, representing an exciting avenue for subsequent studies [36]. In addition, the developed TTNPs can be used to treat other FRα expressing solid tumors such as ovarian, lung and basal breast cancers [37].

## Conclusions

This study presents the successful synthesis and application of a novel PLGA-PEG-pemetrexed copolymer for the targeted delivery of PTX to CRC via the FRα. By covalently linking pemetrexed to the polymer backbone, we developed tumor-targeted NPs (TTNPs) that demonstrated enhanced and selective uptake by FRα-expressing CRC cells both *in vitro* and *in vivo*. Notably, in syngeneic mouse models of CRC, PTX-loaded TTNPs achieved significantly higher tumor accumulation and superior antitumor efficacy compared to non-targeted NPs (NTNPs) and conventional Taxol formulations, without evidence of increased off-target toxicity. These findings underscore the potential of pemetrexed-functionalized NPs to overcome key limitations of conventional chemotherapy by enhancing tumor-specific delivery and therapeutic outcomes in FRα-positive CRC. This work provides a strong foundation for the further development of FRα-targeted nanomedicines and supports the broader applicability of small molecule ligand integration into polymeric delivery systems for targeted cancer therapy. Future investigations will focus on comprehensive pharmacokinetic and toxicological studies, as well as exploring combination regimens with other therapeutic agents, to facilitate clinical translation of this promising platform against FRα expressing cancers.

## Supplementary Information

Below is the link to the electronic supplementary material.

ESM1 (DOCX 90.9 KB)

## Data Availability

The datasets generated during and/or analyzed during this study are available from the corresponding author on reasonable request.

## References

[1] Siegel RL, Wagle NS, Cercek A, Smith RA, Jemal A. Colorectal cancer statistics, 2023. CA Cancer J Clin. 2023;73(3):233–54. 36856579 10.3322/caac.21772

[2] Chibaudel B, Tournigand C, André T, de Gramont A. Therapeutic strategy in unresectable metastatic colorectal cancer. Ther Adv Med Oncol. 2012;4(2):75–89. 22423266 10.1177/1758834011431592 PMC3296081

[3] Wu X, Liu J, Yang L, Wang F. Photothermally controlled drug release system with high dose loading for synergistic chemo-photothermal therapy of multidrug resistance cancer. Colloids Surf B Biointerfaces. 2019;175:239–47. 30540971 10.1016/j.colsurfb.2018.11.088

[4] Singh RK, Patel KD, Mahapatra C, Parthiban SP, Kim TH, Kim HW. Combinatory cancer therapeutics with nanoceria-capped mesoporous silica nanocarriers through ph-triggered drug release and redox activity. ACS Appl Mater Interfaces. 2019;11(1):288–99. 30539634 10.1021/acsami.8b17958

[5] Ngoune R, Peters A, von Elverfeldt D, Winkler K, Pütz G. Accumulating nanoparticles by EPR: a route of no return. J Control Release. 2016;238:58–70. 27448444 10.1016/j.jconrel.2016.07.028

[6] Chen Q, Yuan L, Chou WC, Cheng YH, He C, Monteiro-Riviere NA, *et al*. Meta-analysis of nanoparticle distribution in tumors and major organs in tumor-bearing mice. ACS Nano. 2023;17(20):19810–31. 37812732 10.1021/acsnano.3c04037 PMC10604101

[7] Nakamura Y, Mochida A, Choyke PL, Kobayashi H. Nanodrug delivery: is the enhanced permeability and retention effect sufficient for curing cancer? Bioconjug Chem. 2016;27(10):2225–38. 27547843 10.1021/acs.bioconjchem.6b00437 PMC7397928

[8] Prabhuraj RS, Bomb K, Srivastava R, Bandyopadhyaya R. Selection of superior targeting ligands using PEGylated PLGA nanoparticles for delivery of curcumin in the treatment of triple-negative breast cancer cells. J Drug Deliv Sci Technol. 2020;57:101722.

[9] Tonbul H, Sahin A, Tavukcuoglu E, Esendagli G, Capan Y. Combination drug delivery with actively-targeted PLGA nanoparticles to overcome multidrug resistance in breast cancer. J Drug Deliv Sci Technol. 2019;54:101380.

[10] Wu P, Zhou Q, Zhu H, Zhuang Y, Bao J. Enhanced antitumor efficacy in colon cancer using EGF functionalized PLGA nanoparticles loaded with 5-fluorouracil and perfluorocarbon. BMC Cancer. 2020;20(1): 354. 32345258 10.1186/s12885-020-06803-7 PMC7189558

[11] Tiernan JP, Perry SL, Verghese ET, West NP, Yeluri S, Jayne DG, *et al.* Carcinoembryonic antigen is the preferred biomarker for *in vivo* colorectal cancer targeting. Br J Cancer. 2013;108(3):662–7. 23322207 10.1038/bjc.2012.605 PMC3593555

[12] Ly P-D, Ly K-N, Phan H-L, Nguyen HHT, Duong V-A, Nguyen HV. Recent advances in surface decoration of nanoparticles in drug delivery. Front Nanotechnol. 2024;6:1456939.

[13] Bi D, Zhao L, Yu R, Li H, Guo Y, Wang X, *et al.* Surface modification of doxorubicin-loaded nanoparticles based on polydopamine with pH-sensitive property for tumor targeting therapy. Drug Deliv. 2018;25(1):564–75. 29457518 10.1080/10717544.2018.1440447 PMC6058689

[14] Mochizuki T, Sampei S, Suga K, Watanabe K, Welling TAJ, Nagao D. A quantitative approach to characterize the surface modification on nanoparticles based on localized dielectric environments. Anal Chem. 2024;96(8):3284–90. 38355104 10.1021/acs.analchem.3c03593 PMC10902806

[15] Chen CI, Li WS, Chen HP, Liu KW, Tsai CJ, Hung WJ, *et al*. High expression of Folate receptor alpha (FOLR1) is associated with aggressive tumor behavior, poor response to chemoradiotherapy, and worse survival in rectal cancer. Technol Cancer Res Treat. 2022;21:15330338221141796. 10.1177/15330338221141795 PMC9703519 36426547

[16] D’Angelica M, Ammori J, Gonen M, Klimstra DS, Low PS, Murphy L, *et al*. Folate receptor-α expression in resectable hepatic colorectal cancer metastases: patterns and significance. Mod Pathol. 2011;24(9):1221–8. 10.1038/modpathol.2011.82 21572402

[17] Chattopadhyay S, Moran RG, Goldman ID. Pemetrexed: biochemical and cellular pharmacology, mechanisms, and clinical applications. Mol Cancer Ther. 2007;6(2):404–17. 17308042 10.1158/1535-7163.MCT-06-0343

[18] Adjei AA. Pemetrexed (Alimta): a novel multitargeted antifolate agent. Expert Rev Anticancer Ther. 2003;3(2):145–56. 12722874 10.1586/14737140.3.2.145

[19] Einzig AI, Neuberg D, Wiernik PH, Grochow LB, Ramirez G, O’Dwyer PJ, *et al*. Phase II trial of paclitaxel in patients with advanced colon cancer previously untreated with cytotoxic chemotherapy: an eastern cooperative oncology group trial (PA286). Am J Ther. 1996;3(11):750–4. 10.1097/00045391-199611000-00003 11862233

[20] Kita K, Burdowski A. Recent clinical trials and optical control as a potential strategy to develop microtubule-targeting drugs in colorectal cancer management. World J Gastroenterol. 2024;30(13):1780–90. 38659489 10.3748/wjg.v30.i13.1780 PMC11036503

[21] Ho HN, Tran TH, Tran TB, Yong CS, Nguyen CN. Optimization and characterization of artesunate-loaded chitosan-decorated poly(D,L-lactide-co-glycolide) acid nanoparticles. J Nanomater. 2015;2015(1):674175.

[22] Kazi J, Mukhopadhyay R, Sen R, Jha T, Ganguly S, Debnath MC. Design of 5-fluorouracil (5-FU) loaded, folate conjugated peptide linked nanoparticles, a potential new drug carrier for selective targeting of tumor cells. Medchemcomm. 2019;10(4):559–72. 31057736 10.1039/c8md00565f PMC6482664

[23] Meng F, Wang J, Ping Q, Yeo Y. Quantitative assessment of nanoparticle biodistribution by fluorescence imaging, revisited. ACS Nano. 2018;12(7):6458–68. 29920064 10.1021/acsnano.8b02881 PMC6105334

[24] Li XY, Xu L, Li HL, Du J, Liu XS, Li FH. Au-poly(lactic-co-glycolic) acid complex nanoparticles as ultrasound contrast agents: preparation, characterization and *in vitro* study. Mol Med Rep. 2018;17(3):3763–8. 29286171 10.3892/mmr.2017.8351

[25] Al-natour MA, Abdelrazig S, Ghaemmaghami AM, Alexander C, Kim D-H. Metabolic signatures of surface-modified poly(lactic-co-glycolic acid) nanoparticles in differentiated THP-1 cells derived with liquid chromatography-mass spectrometry-based metabolomics. ACS Omega. 2022;7(33):28806–19. 36033713 10.1021/acsomega.2c01660 PMC9404530

[26] Al-Natour MA, Yousif MD, Cavanagh R, Abouselo A, Apebende EA, Ghaemmaghami A, *et al*. Facile dye-initiated polymerization of lactide-glycolide generates highly fluorescent poly(lactic-co-glycolic acid) for enhanced characterization of cellular delivery. ACS Macro Lett. 2020;9(3):431–7. 10.1021/acsmacrolett.9b01014 35648548

[27] Varshosaz J, Hassanzadeh F, Sadeghi-Aliabadi H, Firozian F. Uptake of etoposide in CT-26 cells of colorectal cancer using folate targeted dextran stearate polymeric micelles. Biomed Res Int. 2014;2014:708593. 24689050 10.1155/2014/708593 PMC3932716

[28] Kwon S, Meng F, Tamam H, Gadalla HH, Wang J, Dong B, *et al*. Systemic delivery of paclitaxel by find-me nanoparticles activates antitumor immunity and eliminates tumors. ACS Nano. 2024;18(4):3681–98. 10.1021/acsnano.3c11445 PMC11025439 38227965

[29] Guerrini L, Alvarez-Puebla RA, Pazos-Perez N. Surface modifications of nanoparticles for stability in biological fluids. Materials. 2018;11(7):1154. 29986436 10.3390/ma11071154 PMC6073273

[30] Croll TI, O’Connor AJ, Stevens GW, Cooper-White JJ. Controllable surface modification of poly(lactic-co-glycolic acid) (PLGA) by hydrolysis or aminolysis I: physical, chemical, and theoretical aspects. Biomacromol. 2004;5(2):463–73. 10.1021/bm0343040 15003007

[31] Mazumdar S, Chitkara D, Mittal A. Exploration and insights into the cellular internalization and intracellular fate of amphiphilic polymeric nanocarriers. Acta Pharm Sin B. 2021;11(4):903–24. 33996406 10.1016/j.apsb.2021.02.019 PMC8105776

[32] Rennick JJ, Johnston APR, Parton RG. Key principles and methods for studying the endocytosis of biological and nanoparticle therapeutics. Nat Nanotechnol. 2021;16(3):266–76. 33712737 10.1038/s41565-021-00858-8

[33] Bertrand N, Wu J, Xu X, Kamaly N, Farokhzad OC. Cancer nanotechnology: the impact of passive and active targeting in the era of modern cancer biology. Adv Drug Deliv Rev. 2014;66:2–25. 24270007 10.1016/j.addr.2013.11.009 PMC4219254

[34] Wang L, Hu D, Xu J, Hu J, Wang Y. Complex *in vitro* model: a transformative model in drug development and precision medicine. Clin Transl Sci. 2023. 10.1111/cts.13695. 10.1111/cts.13695 PMC10828975 38062923

[35] Prabhakar U, Maeda H, Jain RK, Sevick-Muraca EM, Zamboni W, Farokhzad OC, *et al*. Challenges and key considerations of the enhanced permeability and retention effect for nanomedicine drug delivery in oncology. Cancer Res. 2013;73(8):2412–7. 10.1158/0008-5472.CAN-12-4561 PMC3916009 23423979

[36] Zhang J, Wang S, Zhang D, He X, Wang X, Han H, *et al*. Nanoparticle-based drug delivery systems to enhance cancer immunotherapy in solid tumors. Front Immunol. 2023;14:1230893. 10.3389/fimmu.2023.1230893 PMC10435760 37600822

[37] Cheung A, Bax HJ, Josephs DH, Ilieva KM, Pellizzari G, Opzoomer J, *et al*. Targeting folate receptor alpha for cancer treatment. Oncotarget. 2016;7(32):52553–74. 10.18632/oncotarget.9651 PMC5239573 27248175

