# Supplementary material for: Active Targeting of Colorectal Cancer Using Chemotherapy-Loaded Nanoparticles Functionalized with a Folate Receptor-α (FRα) Ligand, Pemetrexed
Source: Pharm Res. 2025 Nov 18;42(12):2339–51. doi: 10.1007/s11095-025-03940-1 (PMC12819448; doi:10.1007/s11095-025-03940-1)
Supplement: Supplementary file 1 — (DOCX 90.9 KB) [file 11095_2025_3940_MOESM1_ESM.docx]

Active Targeting of Colorectal Cancer Using Chemotherapy-loaded Nanoparticles Functionalized with a Folate Receptor-α (FRα) Ligand, Pemetrexed

Mohammad Alnatour^1^, Ramkrishna Sen^1^, Janardhanam Lokesh^1^, Meraj Anjum^1^, Sean Geary^1^, Aliasger K. Salem*^1,2^

^1^ Department of Pharmaceutical Sciences and Experimental Therapeutics, College of Pharmacy, University of Iowa, 180S. Grand Avenue, Iowa City, IA 52242, USA

^2^ Holden Comprehensive Cancer Center, University of Iowa, Iowa City, IA 52242, USA

*Corresponding author: Aliasger K. Salem,

Mailing Address: 452 CPB College of Pharmacy Building (CPB), 180 S. Grand Ave, Iowa City, IA 52242

Telephone number: (319) 335-8810

Email [aliasger-salem@uiowa.edu](mailto:aliasger-salem@uiowa.edu)


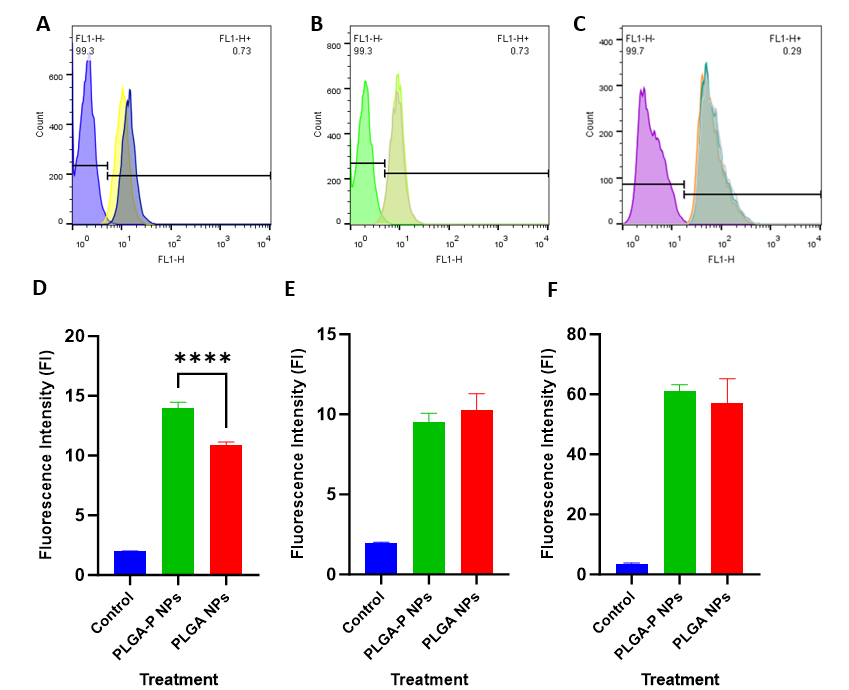


Figure S1: Flow cytometric overlays and statistical analysis of: SW-620 (**A** and **D**) SW-620 with prior 1 mM FA treatment (**B** and **E**), HCT-116 cells (**C** and **F**) Treatment PLGA-P C6 NPs or PLGA C6 NPs at a concentration 10 µg/mL for 1 hour. Cells treated PLGA-P NPs or PLGA NPs were used as a negative control. Data are expressed as the average fluorescence intensity ± SD (n = 3). Statistical analysis was carried out by two-way ANOVA, *** indicates p < 0.001.
